# Disproportionate pace of evidence-based care implementation in pediatric-onset multiple sclerosis: consequences and opportunities

**DOI:** 10.1186/s43058-026-01069-9

**Published:** 2026-08-06

**Authors:** Claudia Gambrah-Lyles, Jordan J. Cole, Alex T. Ramsey

**Affiliations:** 1 Department of Neurology, Division of Pediatric and Developmental Neurology, Washington University School of Medicine, St. Louis, MO 63110 USA; 2 Department of Pediatrics, Section of Neurology, University of Colorado, Aurora, CO USA; 3 Department of Psychiatry, Washington University School of Medicine, 660 South Euclid Ave, St. Louis, MO 63110 USA

**Keywords:** Implementation pace, Implementation speed, Pediatric multiple sclerosis, Transition-to-adult care, Adolescent health

## Abstract

**Background:**

Pediatric-onset multiple sclerosis (POMS) is a chronic autoimmune disease of the central nervous system that requires lifelong care. Long-term outcomes in POMS depend on both early therapeutic intervention and sustained continuity of specialty care into adulthood. However, there is a discrepancy between the speed and consistency at which pharmaceutical disease-modifying therapies and best practices for transition-to-adult care are incorporated into clinical practice. While pharmaceutical disease-modifying therapies have been rapidly incorporated into routine practice, uptake of best practices for transition-to-adult care has been inconsistent and slow, despite evidence-based guidelines and models.

**Main body:**

There is an urgent need for the implementation of transition-to-adult care interventions to “catch up” to that of disease-modifying therapies for POMS. Closing this gap is important because without a system of continuous care, patients lose access to disease-modifying therapies, negating the benefits of early and rapid uptake and jeopardizing long-term neurologic stability and outcomes as youth with multiple sclerosis (MS) grow into adulthood. This paper applies the Framework to Assess Speed of Translation (FAST) to examine this contrast in implementation pace and understand why pharmaceutical disease-modifying therapies have diffused rapidly, whereas innovations to support transitioning to adult care have not. We examine how innovation, adopter and contextual factors affect the diffusion and uptake of innovations in POMS, illustrating how interventions with observable relative advantage, strong champions, and compatibility with existing medical paradigms are propelled through the system, whereas interventions that are perceived as more complex, diffuse in ownership, and less visibly linked to outcomes are hindered. We argue that future efforts to improve transition-to-adult care in POMS must systematically identify and address determinants of pace that have slowed progress to date. Guided by the Designing for Accelerated Translation (DART) framework, we propose strategies for developing and mapping transition-to-adult care interventions that emphasize rapid translation.

**Conclusion:**

Our objective is to facilitate the acceleration of translation of evidence-based transition-to-adult care practices in POMS so that continuity of care keeps pace with therapeutic innovation, maximizing long-term outcomes for this population.

Contributions to the literature

- Advances understanding of implementation pace by examining unequal diffusion of two innovations – disease-modifying therapies and transition-to-adult care practices – in pediatric-onset multiple sclerosis. Uses this contrast to demonstrate how determinants of implementation pace play out in real-world settings and positions speed as an actionable target for improving care delivery.
- Explicitly applies two frameworks focused on implementation pace to a clinical domain and embeds speed, ownership, and contextual fit into development of transition-to-adult care innovations.
- Although grounded in pediatric-onset multiple sclerosis, proposes an agenda that can inform efforts to accelerate transition practices beyond a single disease to other chronic childhood conditions.

## Introduction

An estimated 2.8 million people worldwide have multiple sclerosis (MS) [1], a chronic central nervous system disease causing relapsing neurological impairment and neurodegeneration. Up to 10% of cases begin in childhood [2], and compared to adult-onset MS, pediatric-onset multiple sclerosis (POMS) has a more aggressive onset [3], higher relapse rate [3], and earlier cognitive and physical disability [4]. MS has no cure, but advances in MS pathogenesis have produced multiple pharmaceutical disease-modifying therapies that mitigate disease [5]. Starting from only two treatments in the early 1990s, there are now more than 20 FDA-approved MS disease-modifying therapies [5]. 

Implementation science often cites a 15 to 17-year gap from evidence generation to clinical use [6], yet rapid development and uptake of disease-modifying therapies in MS represents a striking exception. The average time from clinical trial publication to FDA approval across all 20 disease-modifying therapies is 1.13 years, and population-based studies show widespread use within 5 years of FDA approval [7]. This phenomenon extends to POMS, where disease-modifying therapies have been rapidly implemented despite limited trial data in children. [7, 8].

Although prompt uptake of disease-modifying therapies in POMS has led to better disease control [8], effective management must also address continuity of care as children age into adulthood [9]. For children with chronic disease, the move to adult care is often associated with inappropriate healthcare utilization, reduced treatment adherence, and negative health outcomes [9], underscoring the need for evidence-based transition-to-adult care practices that promote continuous care in the adult healthcare system.

Transition to adult care is defined as a structured process that prepares young adults for adult-oriented healthcare [9]. Structured transition-to-adult care programs are associated with improved health outcomes, care continuity, and increased patient satisfaction [9]. Despite these benefits, survey data from ten POMS clinics in the United States reveals variable transition-to-adult care practices and lack of adherence to evidence-based recommendations [10]. Reflective of this gap, POMS patients report feeling unprepared for the transition process [11], and they experience poor medication adherence and increased disability after leaving pediatric care [12]. POMS is a chronic condition with unique and ongoing healthcare needs, and effective management requires a well-coordinated transition-to-adult care process.

POMS presents a unique opportunity to understand factors influencing the pace of implementation given this existing dichotomy between rapid uptake of disease-modifying therapies and slow adoption of structured transition-to-adult care practices. Using the Framework to Assess Speed of Translation (FAST) [13], we examine why advancements in disease-modifying therapy have been swiftly integrated while transition-to-adult care practices have not. In doing so, we identify key aspects of transition-to-adult care practices that may be specifically targeted to improve speed of uptake.

As young people with MS grow into adulthood, the widening gap between translation of disease-modifying therapies and translation of interventions that promote healthcare continuity threatens to undermine long-term outcomes. We argue that future efforts to improve transition-to-adult care in POMS must focus on prioritizing factors and characteristics that specifically promote speed of uptake of these practices.

### Contrasting the speed of uptake of innovations in POMS: application of the framework to assess speed of translation (FAST)

The FAST framework draws on Rogers’ Diffusion of Innovations Theory [14] to provide a scaffold for examining factors that influence an innovation’s implementation speed. FAST primarily outlines three core determinants – the innovation itself, adopters, and contextual factors. Examining these domains as applied to the two innovations of interest – disease-modifying therapies and transition-to-adult care practices in POMS – illuminates key contrasts in their perception and integration in practice, providing theory-based explanations for their divergent implementation speeds. Of note, both “disease-modifying therapies” and “transition-to-adult care practices” could perhaps more accurately be described as innovations that encompass multiple distinct evidence-based interventions, with examples provided in Table 1. Furthermore, many tenets of transition-to-adult care practices, and their related interventions, are broadly applicable across childhood-onset chronic disease, but condition-specific recommendations exist as well. Though there are potential unique features regarding speed of implementation *within* each group, the differences *between* groups are much more striking, and are the focus of this analysis.

**Table 1:** Examples of discrete evidence-based innovations within disease modifying therapies and transition-to-adult care practices

Disease-Modifying Therapies	Function	Transition-to-Adult Care Practices	Function
**B-Cell Depleting Therapies** (e.g. Ocrelizumab)	Reduces immune cells that drive MS damage; highest efficacy, used across disease stages	**Transition Policy/Guide**	Establishes formal frameworks, expectations, and roles for transition
**S1P Modulators** (e.g. Fingolimod)	Keeps immune cells from reaching the brain/spinal cord; may be limited by adherence	**Transition Readiness Assessment**	Systematically evaluates patient preparedness for adult care
**Alpha integrin blockade** (e.g. Natalizumab)	Prevents immune cells from entering the brain; high risk profile and monitoring need	**Individual Skills Training**	Builds patient capacity for independent disease management
**Fumarates** (e.g.Dimethyl Fumarate)	Calms immune overactivity; selection influenced by tolerability & pregnancy plans	**Patient Tracking and Monitoring**	Ensures continuity through structured follow-up during transfer
**Glatiramer Acetate**	Redirects immune response; often used when safety in pregnancy or long-term use is prioritized	**Medical Summarization/ History**	Provides a standardized clinical summary to support safe handoff

Table 1: Key disease modifying therapies used in multiple sclerosis are listed in the first column. Evidence-based transition-to adult care practices as defined by the Six Core Elements of Healthcare Transition are listed in the second column

### FAST construct I: the innovation

**Fig. 1:**
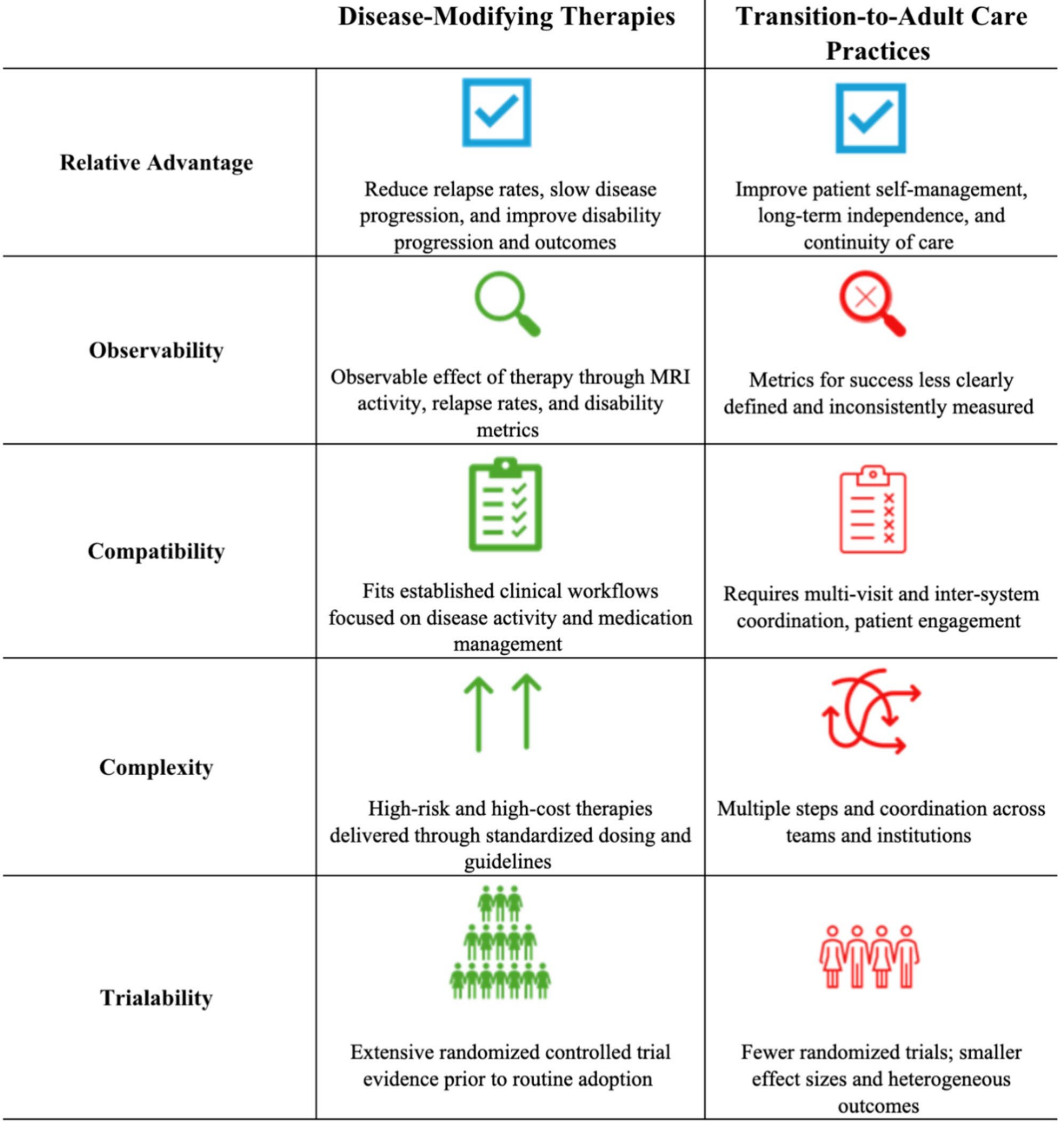
Innovation characteristics of disease-modifying therapies and transition-to-adult care practices. Conceptual comparison of innovation characteristics influencing adoption of disease-modifying therapies and transition-to-adult care practices, adapted from diffusion of innovations theory

The Diffusion of Innovations Theory identifies five key attributes (relative advantage, observability, compatibility, complexity, and trialability) of an innovation that contribute to its future success. Figure 1 and the following text compare innovation characteristics between disease-modifying therapies and transition-to-adult care practices.

### Disease-modifying therapies

Disease-modifying therapies demonstrate strong adoption potential across all five innovation attributes. They offer a clear and observable relative advantage by reducing relapse rates, slowing disease progression, and improving measurable disability outcomes [8]. Disease-modifying therapies align with existing clinical paradigms, which prioritize pharmacological treatment and quantifiable disease control. Although complex and associated with cost and risk, they are operationalized through standardized dosing and established guidelines [15]. They have been evaluated in large, randomized trials using familiar endpoints; [15] and at the clinician level, an individual neurologist can trial disease-modifying therapy in one or a few patients during routine care without needing additional training or other staff involvement. Critically, their effects are highly observable: MRI activity, relapse rates, and disability metrics provide measurable, objective feedback on treatment impact [15]. 

### Evidence-based transition-to-adult care practices

In contrast, transition-to-adult-care innovations have less favorable innovation characteristics. Although their relative advantage is supported by evidence linking structured transition processes to improved outcomes [9], these benefits are less immediately observable. Benefits such as improved self-management and long-term care continuity are primarily indirect and long-term. We hypothesize that these outcomes are poorly captured by the short-term, disease-focused metrics that medical systems typically use to evaluate clinical success, which may limit their perceived value.

Compatibility with clinical practice patterns is also limited. Transition-focused interventions emphasize psychosocial readiness, autonomy, and skillset development [9] – concepts that cannot be met within a single visit already constrained by competing clinical demands [16]. Transition-to-adult care practices are also perceived as highly complex [16], requiring coordination across pediatric and adult providers and institutional support. Furthermore, trialability is constrained by very few randomized controlled trials [17], often with short follow-up duration and heterogeneous endpoints. Observability is further compromised because metrics for success have not been well-defined [17], and lastly those who must initiate transition-to-adult are innovations (i.e., POMS clinicians and clinic staff) are inherently unlikely to observe the downstream impacts of such innovations after a patient transfers to the adult setting.

### FAST construct II: adopters

FAST emphasizes that implementation speed is shaped by adopter characteristics and dynamics across multiple levels. Table 2 compares these relationships across provider, patient/family, and organizational levels for disease-modifying therapies and transition-to-adult care practices and highlights how the presence of a clearly defined primary adopter, or champion, strengthens alignment across levels and accelerates uptake, whereas diffuse responsibility slows implementation.

**Table 2:** Adopter characteristics - disease modifying therapies vs. transition-to-adult care practices

	Disease-Modifying Therapies	Transition-to-Adult Care Practices
Primary Adopter	Clearly Defined: A prescribing clinician holds decision authority and accountability [16]	Not Clearly Defined: No single role consistently holds responsibility for leading or sustaining transition [10,17]
Provider	Treatment adoption and initiation are core clinical responsibilities, [16] concentrating authority and expertise within the clinician role	Transition planning and adoption are not consistently anchored within the provider role and compete with other clinical priorities [10,17]
Patient / Family	Adoption occurs within a clinician-directed treatment structure motivated by patient desire for relapse prevention, limiting disability, and improved quality of life [5,8,16]	Adoption is motivated by recognition of transition as essential for long-term independence [12] but is constrained when preparation for adult care is not consistently structured or led [10,17]
Organizational	Adoption occurs due to benefits that are measurable, readily observable, and linked to a clearly accountable role [5,8,16,19]	Adoption is constrained when benefits are longer-term, difficult to measure, and not linked to a single accountable role [10,18,19]

Comparison of adopter characteristics for disease-modifying therapies and transition-to-adult care practices, organized by adopter level (provider, patient/family, and organizational)

### Disease-modifying therapies

For POMS disease-modifying therapies, the primary innovation adopters are pediatric neurologists and MS specialists who ultimately control what treatment is prescribed for POMS patients. Patients and families, who must agree to and follow through with therapy when prescribed, are adopters of this process, and organizational leaders who shape the policies and resources that enable access to disease-modifying therapies are adopters as well. Because authority for initiating disease-modifying therapy rests with the provider [15], adoption of therapy is organized around a discrete treatment decision. Patient motivations to prevent relapses, limit disability, and improve quality of life [18] align with that decision, and organizational priorities favor measurable improvements in disease control that are reflected in routine clinical performance metrics [19]. This concentrated ownership strengthens alignment across adopter levels and promotes rapid uptake and diffusion.

### Evidence-based transition-to-adult care practices

In contrast, transition-to-adult care practices lack a clearly defined primary adopter. Responsibility for initiating and sustaining transition-to-adult care practices is unclear and distributed across roles without a shared framework [9, 16]. For example, transition planning is not consistently anchored within the provider role and is often deprioritized amid competing clinical demands [16]. Although patients and families recognize the importance of this preparation, no single role consistently guides increasing autonomy [11, 16]. At the organizational level, transition outcomes are longer-term and currently not easily measured [9, 17], making them harder to consistently resource and prioritize [19]. Thus, while providers, patients/families, and organizations all function as adopters in this context, presenting an opportunity for shared ownership, in the absence of clearly defined responsibilities none hold concentrated authority or accountability. This diffuse ownership weakens cross-level alignment and slows uptake of transition-to-adult care practices.

### FAST construct III: contextual factors

The adoption of innovations is inherently affected by contextual factors that also may affect pace, such as time-sensitive public health emergencies, resource funding and availability, and implementation infrastructure.

### Disease-modifying therapies

For POMS, the uptake of disease-modifying therapies has been accelerated by urgency due to disease severity. Compared to adult-onset MS, POMS is characterized by more aggressive onset [3], higher relapse rate [3], and earlier cognitive and physical disability [4], creating an urgent need for effective treatment options [8]. Even in the absence of many pediatric-specific randomized controlled trials [18], POMS presentation and disease course have generated a strong sense of therapeutic urgency and justified rapid diffusion of disease-modifying therapy use - at times exceeding uptake rates in adult populations [7, 8]. 

The urgency to mitigate disease severity has been further reinforced by a funding landscape primed to expedite implementation of emerging pharmacologic innovations. Although the high costs of disease-modifying therapies have prompted payer restrictions, public and private insurers have ultimately provided coverage for many treatments [5], supported by evidence that disease-modifying therapy use and adherence are associated with fewer relapses and lower hospitalization rates [5, 8, 15]. Furthermore, the healthcare system was already structured to facilitate uptake of disease modifying therapies. Existing infusion services, neurology specialty clinics, and pharmacy infrastructure for specialty drugs positioned providers to deliver disease-modifying therapies with minimal operational disruption.

### Evidence-based transition-to-adult care practices

Conversely, contextual factors such as ambiguity in timing, limited financial incentives, and structural constraints have limited the diffusion of transition-to-adult care innovations. Unlike pharmacologic decisions – which typically have discrete clinical triggers [15] – there is no universally defined transition point for patients [9]. While guidelines recommend that transition conversations begin at a certain age or developmental stage [9], in practice, transition is shaped by relational and developmental factors rather than a single milestone. For example, surveyed providers report that patient age, perceived “readiness,” and complexity of the adolescent’s needs all play a role in decisions to transition the patient [16]. 

In addition, organizational limitations compound this variability. Adult provider availability, expertise and institution specific policies all influence whether formal transition programs can be sustained [9]. In many settings, adult services are already constrained; pediatric clinicians lack dedicated time for transition planning; and existing workflows are not designed to support cross-team coordination, leading to delayed or absent transitions [9, 16]. Economic and incentive structures further constrain uptake. A billing code for pediatric-to-adult transition counseling exists [9], but there is little evidence that it is routinely or effectively used to support implementation. Unlike disease-modifying therapies - which have clear revenue and reimbursement infrastructure [5] - transition-to-adult care interventions primarily rely on care coordination that is not directly tied to predictable or sustainable funding streams [9, 16]. These limitations are also magnified by system-level barriers such as fragmented healthcare systems that disrupt continuity and weaken the foundation for sustained care [9, 16]. 

### Consequences of disproportionate diffusion: accelerated therapeutic uptake without coordinated transition

Disease-modifying therapy uptake in POMS represents how an innovation with strong attributes, clear adopters, and contextual urgency achieves rapid and efficient uptake, flowing smoothly through the diffusion pipeline. In contrast, uptake of transition-to-adult care practices represents a leaky and fragmented pipe; weaker innovation attributes, the absence of clear adopters, and complex contextual challenges constrain diffusion, resulting in progressively reduced uptake.

**Fig. 2:**
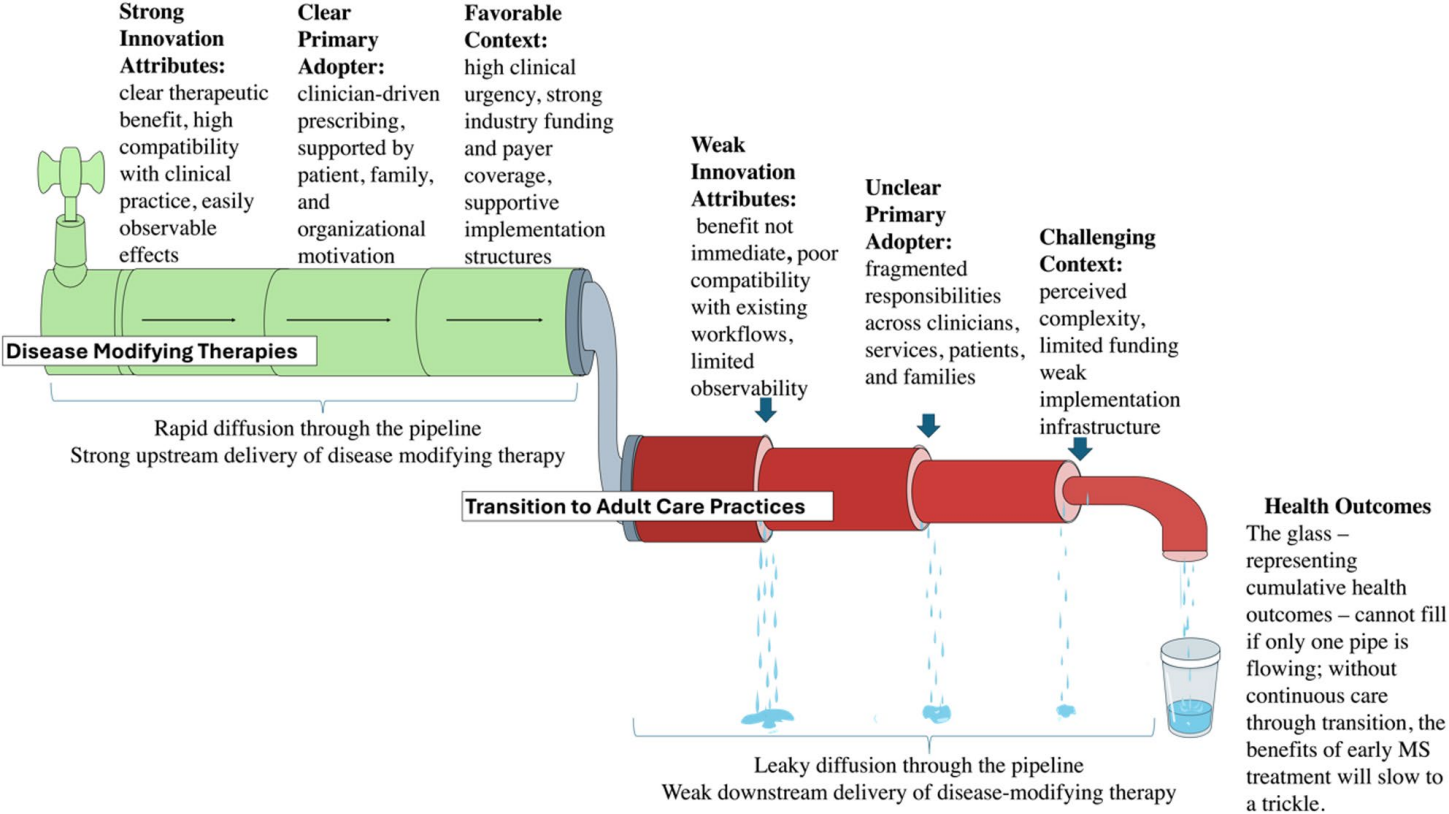
Uneven Diffusion of Innovation in POMS. Conceptual model of pipeline of care in pediatric-onset multiple sclerosis: despite strong diffusion and delivery of disease-modifying therapies, weak uptake of transition-to-adult care restricts how much benefit ultimately reaches patients by adulthood

Figure 2 illustrates this connected pipeline and demonstrates how, despite strong upstream diffusion and delivery of disease-modifying therapies, poor uptake of transition-to-adult care practices limits how much of that benefit ultimately reaches patients by adulthood. Critically, the glass - representing cumulative patient health outcomes - can never remain full if only one pipe is functioning appropriately.

Without a system of continuous care, young people with MS risk losing access to disease-modifying therapies, inhibiting the benefits of early and rapid uptake, and leading to worse long-term outcomes. As young people with MS grow into adulthood, there is an urgent need for the uptake of transition-to-adult care interventions to “catch up” to that of disease-modifying therapies to ensure uninterrupted flow of benefit across the care continuum. Accordingly, guided by the Designing for Accelerated Translation (DART) framework [20], we will now shift attention from describing barriers to proposing strategies that enable better uptake and diffusion of transition-to-adult care practices.

### Improving the speed of translation of transition-to-adult care practices in POMS: Application of the designing for accelerated translation (DART) framework

Given the above-detailed inhibitors to uptake of transition-to-adult care practices, and guided by the contrasting accelerators of disease-modifying therapies, we now utilize the DART framework to answer the following question: *How might modifiable features of transition-to-adult care practices be enhanced or adjusted to increase pace of implementation?*

DART emphasizes that translation speed depends not only on evidentiary strength, but also on *demand* (clinical need), *risk* (harms of action or delay), and *cost* (resources required for implementation). It proposes a three-pronged approach to accelerating the uptake of innovations: learning health systems, meaningful stakeholder partnership, and designing for dissemination and implementation [20]. 

**Fig. 3:**
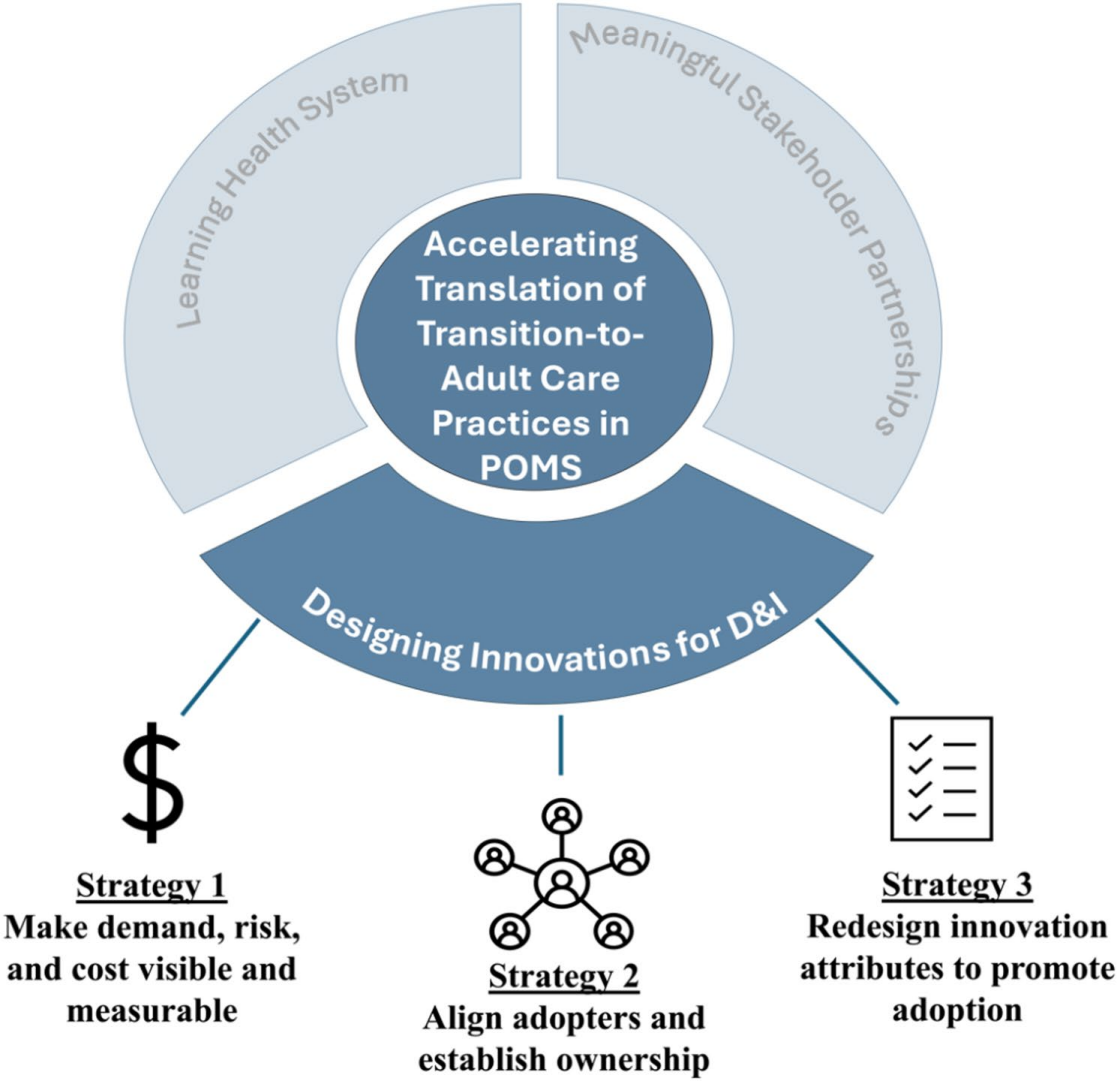
Research strategies designed to accelerate the dissemination and implementation of Transition-to-adult care practices in POMS based on DART framework. Figure adapted from Ramsey et al20

In this work, we concentrate on the third of these approaches – designing for dissemination and implementation – the DART strategy that involves intentionally shaping an innovation to fit the environment in which it must operate. Transition-to-adult care practices for POMS have lacked features that drive uptake, and a pragmatic, speed-focused approach is necessary to address these limitations. We propose three strategies to redesign transition-to-adult care practices to promote more rapid implementation in the context of POMS (Fig. 3).

### Redesign strategy 1: make demand, risk, and cost visible and measurable

Transition outcomes in POMS have not been consistently reported [12], making the need for transition-to-adult care practices hard to demonstrate. Future work should quantify treatment interruptions, preventable relapses, and loss to follow-up during and after transfer. Clinical consequences of these disruptions – including disability progression and acute care utilization – should be explicitly measured. Studies should also characterize the operational burden of delivering transition support, examine financial value of transition-related billing codes, and quantify how continuous access to disease-modifying therapy prevents relapse and offsets downstream healthcare costs. Making demand, risk, and cost visible in this way would strengthen the case for transition-to-adult care practices to be recognized and supported as necessary elements of POMS management.

### Redesign strategy 2: redesign innovation attributes to promote adoption

Transition-to-adult care practices must be designed for use within existing POMS care structures. Redesign efforts should explicitly target the innovation attributes that are known to accelerate adoption:

- **Highlight Relative Advantage**: Emphasize how transition-to-adult care practices not only improve self-management but also work to prevent disease worsening by maintaining access to disease-modifying therapy.
- **Increase Compatibility**: Integrate transition-to-adult care tasks (e.g., readiness assessments, referral triggers, documentation) into standard neurology workflows and electronic health records so that transition support occurs alongside standard clinical care.
- **Reduce complexity**: Streamline intervention requirements by using brief, standardized assessments that can be completed during usual visits.
- **Improve Trialability**: Evaluate transition approaches in formats that allow small-scale piloting (e.g., stepped-wedge designs, quality improvement PDSA cycles), enabling teams to test and refine processes within their usual operations.
- **Enhance Observability**: Utilize and track intervention outcomes that are directly tied to clinical goals, such as relapse prevention and engagement with adult MS care and provide this information back to pediatric teams so improvements are visible.

### Redesign strategy 3: align adopters and establish accountable ownership

Variation in who initiates and completes transition-to-adult care practices slows diffusion and uptake in POMS. Redesign efforts should identify who is responsible for each transition task and ensure these roles fit within existing neurology workflows. At the organizational level, adoption should include secured funding and resources and policies that consider insurance coverage and service continuity during transfer.

These three redesign strategies translate DART principles into concrete research directions for POMS. By strengthening value, usability, and ownership within existing systems, they lay the groundwork for future interventions in transition-to-adult care that can be adopted rapidly and reliably across pediatric MS care settings.

## Conclusion

Rapid therapeutic progress in POMS has not been matched by progress in ensuring continuous care through adulthood. Our application of the FAST framework demonstrates that weak innovation attributes, unclear adopters, and inhibitory contextual factors limit the diffusion and uptake of transition-to-adult care practices. Guided by the DART framework, we propose a pragmatic research agenda for targeting these determinants of implementation speed to close this gap, preserve early treatment benefits and support youth with MS in achieving healthier, more stable futures in the adult healthcare system.

## Data Availability

Not applicable. Data sharing is not applicable to this article as no datasets were generated or analysed during the current study.
